# Dietary and lifestyle factors associated with troublesome gastroesophageal reflux symptoms in Vietnamese adults

**DOI:** 10.3389/fnut.2023.1280511

**Published:** 2023-11-08

**Authors:** Duc Trong Quach, Mai Ngoc Luu, Phong Van Nguyen, Uyen Pham-Phuong Vo, Cong Hong-Minh Vo

**Affiliations:** ^1^Department of Internal Medicine, University of Medicine and Pharmacy at Ho Chi Minh City, Ho Chi Minh City, Vietnam; ^2^Department of Gastroenterology, Nhan Dan Gia Dinh Hospital, Ho Chi Minh City, Vietnam

**Keywords:** gastroesophageal reflux disease, Vietnamese, diet, lifestyles, risk factors

## Abstract

**Background:**

Dietary and lifestyle habits related to troublesome gastroesophageal reflux symptoms (tGERS) differ significantly across populations. There have yet to be studies on the Vietnamese population.

**Aims:**

To identify dietary and lifestyle habits associated with tGERS in Vietnamese adults.

**Methods:**

A cross-sectional survey was conducted among Vietnamese adults aged 18 years and older from March 2023 to May 2023. Participants were recruited online through a widely read national newspaper and Facebook, one of Vietnam’s most popular social media platforms. The survey questionnaire comprised 27 questions covering participants’ basic demographic information, dietary and lifestyle habits, the presence and characteristics of tGERS, and inquiries about specific dietary and lifestyle patterns, foods, and beverages associated with tGERS.

**Results:**

A total of 4,400 valid responses were collected, including 2050 participants without tGERS and 2,350 participants with tGERS. Multivariate analysis showed several factors associated with tGERS, including eating beyond fullness (OR 1.383, CI95% 1.127–1.698), tight clothing (OR 1.627, CI95% 1.256–2.107), stress (OR 1.566, CI95% 1.363–1.800), and insomnia (OR 1.321, CI95% 1.129–1.546). Among habits associated with tGERS, eating beyond fullness was the most frequently reported (64.6%). Interestingly, although a short meal-to-bed time and staying up late after midnight were not risk factors for tGERS, they were two common factors associated with tGERS in symptomatic participants, particularly those with nocturnal reflux symptoms. For food triggers, the three most common ones were greasy foods (71.9%), sour/spicy soups (64.7%), and citrus fruits (36.0%). In terms of beverages, carbonated soft drinks were at the top of triggering tGERS (40.3%), and beer and orange juice were the second and third most common triggers, accounting for 35.7 and 30.6%, respectively.

**Conclusion:**

We reported the dietary and lifestyle habits associated with tGERS in Vietnamese adults for the first time. These findings will serve as a basis for future studies on the primary prevention and nondrug management of tGERS in Vietnam.

## Introduction

Gastroesophageal reflux disease (GERD) is a prevalent upper gastrointestinal disorder worldwide (1). Although it was once thought to be more common in Western countries, its prevalence tends to increase in Asia, particularly in Southeast Asia (2). The present situation is partly explained by dietary and lifestyle changes and an increase in body mass index (BMI), particularly in conjunction with dramatic socioeconomic development in this region (3). Heartburn and regurgitation are the cardinal symptoms of GERD (4). As the definitive diagnosis of GERD requires objective evidence based on endoscopy or functional investigations that are not widely available, the presence of troublesome gastroesophageal reflux symptoms (tGERS) is commonly used to suggest GERD diagnosis in daily practice (4, 5).

Approximately more than two-thirds of GERD cases are nonerosive reflux diseases (2). Among those with endoscopic esophageal injuries, the majority have mild-grade reflux esophagitis, and complications are infrequent, especially in Asian populations (2). Although GERD is not life-threatening, it tends to recur and significantly affects patients’ quality of life (6). Therefore, symptom control is considered a priority in GERD management. Dietary and lifestyle modifications are the first-line therapy recommended in all current guidelines, despite low evidence (4, 7, 8). Notably, dietary and lifestyle habits associated with tGERS vary dramatically across populations (9). Routine recommendations to modify various dietary and lifestyle habits can decrease patients’ quality of life and compromise their long-term adherence. Therefore, it is necessary to conduct additional studies to assess the risk factors for tGERS and patients’ feedback on specific foods and beverages that trigger tGERS in each population. Such data would help physicians with primary prevention strategies and effective counseling.

Vietnam is a developing country in Southeast Asia with a population of nearly 100 million, and it has diverse cuisines. The prevalence of GERD in Vietnam has been increasing over the past 20 years, and it is now one of the most common upper gastrointestinal disorders (10). Although there have been some studies on GERD in Vietnam, most previous studies are hospital-based, and there have been no population-based studies on the association between dietary and lifestyle habits and tGERS. This study aimed to (1) identify dietary and lifestyle factors associated with tGERS and (2) record the feedback of Vietnamese adults with tGERS on the specific dietary and lifestyle habits, foods, and beverages that associated with tGERS.

## Materials and methods

### Study design and participants

A cross-sectional online survey was conducted via a widely read national online newspaper and Facebook, one of most popular social media platforms in Vietnam, from March 2023 to May 2023. The survey targeted the general Vietnamese population aged 18 years or above. The questionnaire was built on the web platform https://www.surveymonkey.com/. The consent form was presented on the first page of the survey link. By clicking the “Agree” button on this page, the participants agreed to participate and would complete the survey online. Confidentiality and anonymity were maintained, as no individually identifiable information was obtained during the questionnaire.

The survey was carried out using a convenience sample and included any adult participants who completed the survey questionnaire. Responses were excluded from the analysis if the respondents failed to answer all survey questions completely or the completion time was unreliable according to the software’s estimated finishing time. In addition, responses with duplicated internet protocol addresses were also excluded.

Ethics approval was obtained for this study (numbered 011/NDGD-HDDD, signed on March 22, 2023).

### Development of the survey questionnaire

One senior investigator with more than 20 years of experience in GERD management (DTQ) drafted the questionnaire. Questions about dietary and lifestyle habits and specific foods and beverages that associated with tGERS were developed based on his expertise in managing GERD in Vietnamese. They were also referenced from a recent systematic review of the dietary and lifestyle factors related to tGERS (9). The other four research team members then pretested the initial survey questionnaire to verify the clarity and comprehension of each question. Following this, a pilot test was conducted on 30 individuals experiencing reflux symptoms to examine the survey’s reliability. The reliability was assessed using Cronbach’s alpha coefficient, which yielded a value of 0.61, indicating an acceptable internal consistency (11). In addition, the content validity of the survey questionnaire was evaluated using the content validity index, resulting in an index of 0.81, which indicates a good content validity (12, 13). Subsequent adjustments were made as necessary after this evaluation. The final survey questionnaire comprised three sections (Supplementary material S1). The first section collected demographic data, including age, sex, height, weight, education level, current place of residence, and current job. The second section gathered information on dietary and lifestyle habits, including staying up late after midnight, skipping breakfast, eating within 2 h before bed, eating beyond fullness, wearing tight clothing, smoking, anxiety, and insomnia. They were rated on a 5-point Likert frequency scale (never, rarely, occasionally, fairly often, and very often). The third section of the questionnaire determined about the presence and characteristics of tGERS. tGERDS include esophageal and extra-esophageal ones. However, the symptoms which supposed to be extra-esophageal are often non-specific and can be caused by various otorhinolaryngological, maxillofacial and respiratory diseases (4, 8). Therefore, in this survey we only address the typical reflux symptoms (i.e., hearburn and regurgitation). Participants who did not have tGERS within the last month finished the survey after answering the first two sections of the questionnaire. Those with tGERS continued to answer questions about tGERS frequency, the timing of symptom occurrence and symptom duration. The participants were also asked about their self-management methods, medical consultations, and adherence to adjusting their diet and lifestyles. These questions were in multiple-choice, single-select format. In addition, the lifestyles, eating habits, foods, and beverages that associated with tGERS were recorded. For these questions, they could choose multiple options that suited them and/or state other options. Within the scope of this study, we only investigated the factors associated with tGERD but not GERD, the disease. Therefore, objective investigations for GERD diagnosis such as upper gastrointestinal endoscopy and 24 h esophageal pH monitoring measurement were not applied.

### Statistical methods

All statistical analyses were performed with SPSS 23 (SPSS Inc., Chicago, IL). Categorical variables are presented as numbers and percentages and were compared using Pearson’s chi-squared test. Continuous variables were tested for normality using the Kolmogorov–Smirnov test. Those with a nonnormal distribution are presented as the median (upper and lower quartiles) and were compared using the Mann–Whitney U test. Univariate logistic regression was performed to identify factors associated with tGERS. The dietary and lifestyle habits assessed on the Linkert score were divided into two groups for comparison: group 1 (never, rarely, or occasionally) versus group 2 (fairly often or very often). Variables with *p* values <0.2 in univariate analysis were used for multiple logistic regression analysis. All *p* values were two-sided, and those less than 0.05 were considered statistically significant.

## Results

### Participant characteristics

**Figure 1:**
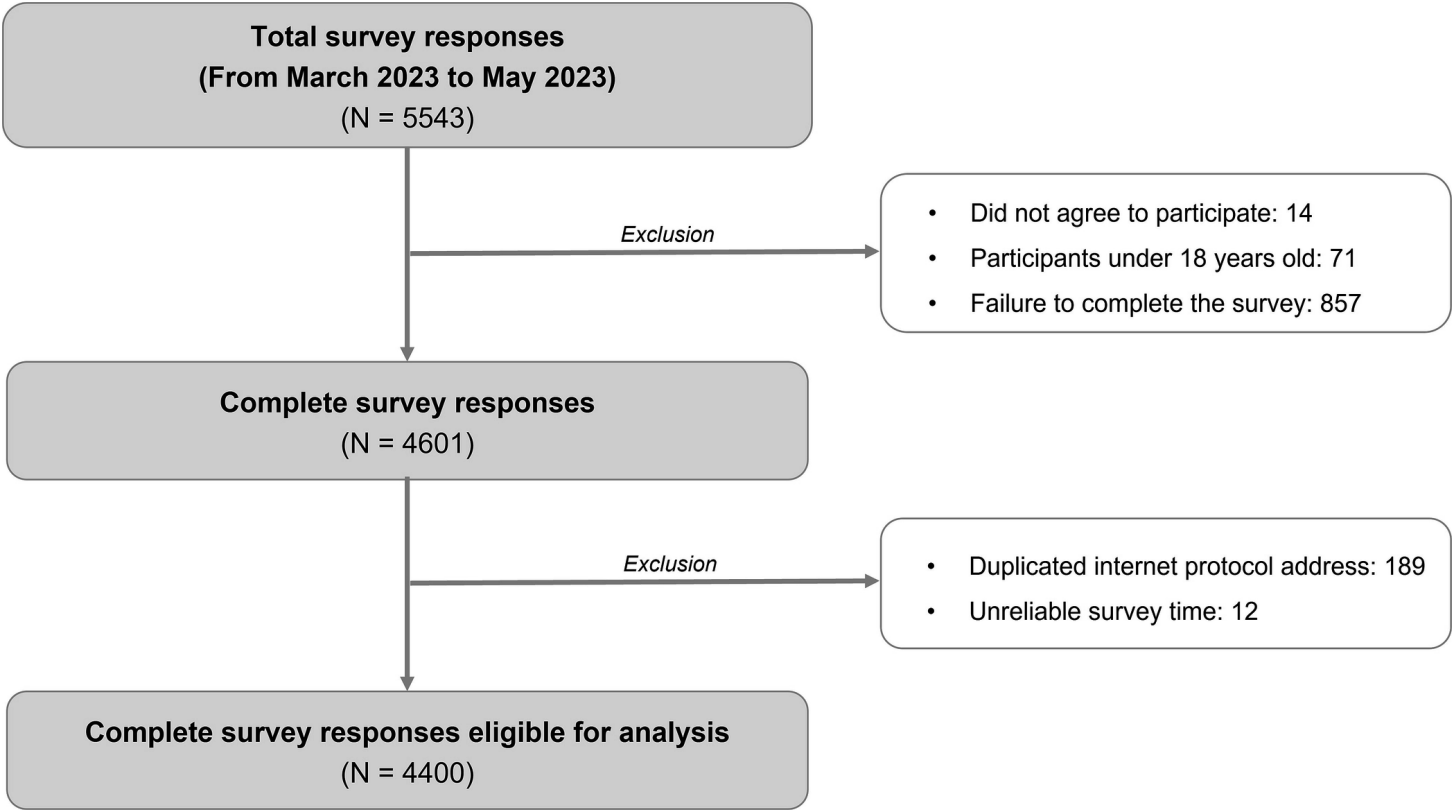
Recruitment flowchart of participants in the survey.

There were 4,400 valid responses from March 2023 to May 2023, including 2050 responses from participants without troublesome reflux symptoms and 2,350 responses from those with at least one troublesome reflux episode within the past month (Figure 1). The participants’ demographic characteristics are presented in Table 1.

**Table 1:** Participants’ demographic characteristics.

Characteristics	Participants without tGERS (*N* = 2,050)	Participants with tGERS (*N* = 2,350)	Total (*N* = 4,400)	*p*
**Gender**				0.185
Male	470 (22.9)	592 (25.2)	1,062 (24.1)	
Female	1,577 (66.9)	1,753 (74.6)	3,330 (75.7)	
Other^a^	3 (0.1)	5 (0.2)	8 (0.2)	
**Age**				<0.001
18–30	711 (34.7)	679 (28.9)	1,390 (31.6)	
31–40	768 (37.5)	897 (38.2)	1,665 (37.8)	
41–50	344 (16.8)	507 (21.6)	851 (19.3)	
51–60	169 (8.2)	195 (8.3)	364 (8.3)	
Over 60	58 (2.8)	72 (3.1)	130 (3.0)	
**Height (cm)** (median, IQR)	158 (155–164)	158 (155–164)	158.0 (155–164)	0.900
**Weight (kg)** (median, IQR)	54 (49–62)	55 (49–63)	55.0 (49–62)	0.008*
**BMI** (median, IQR)	21.6 (19.9–23.5)	21.9 (20.0–24.1)	21.7 (20.0–23.9)	<0.001*
**Level of education**				0.012
Primary/secondary/high school	192 (9.4)	217 (9.2)	409 (9.3)	
College/university	1,478 (72.1)	1,613 (68.6)	3,091 (70.2)	
Post-graduate	380 (18.5)	520 (22.1)	900 (20.5)	
**Domicile**				0.347
Urban	1,670 (81.5)	1940 (82.6)	3,610 (82.0)	
Rural	380 (18.5)	410 (17.4)	790 (18.0)	
**Career**				0.017
Office work	1,356 (66.1)	1,651 (70.3)	3,007 (68.3)	
Freelance trading/business	241 (11.8)	262 (11.1)	503 (11.4)	
Transportation – delivery	13 (0.6)	24 (1.0)	37 (0.8)	
Agriculture/manual labor	43 (2.1)	43 (1.8)	86 (2.0)	
Student	138 (6.7)	114 (4.9)	252 (5.7)	
Retirement	34 (1.7)	29 (1.2)	63 (1.4)	
Housewife	225 (11.0)	227 (9.7)	452 (10.3)	
**Habits (fairly or very often)**				
Staying up late after midnight	521 (25.4)	641 (27.3)	1,162 (26.4)	0.162
Skipping meals	361 (17.6)	426 (18.1)	787 (17.9)	0.655
Eating within 2 h before bed	692 (33.8)	855 (36.4)	1,547 (35.2)	0.069
Eating beyond fullness	179 (8.7)	306 (13.0)	485 (11.0)	<0.001
Wearing tight belongings	98 (4.8)	194 (8.3)	292 (6.6)	<0.001
**Smoking**	58 (2.8)	68 (2.9)	126 (2.9)	0.898
**Stress (fairly or very often)**	573 (28.0)	935 (39.8)	1,508 (34.3)	<0.001
**Insomnia (fairly or very often)**	402 (19.6)	649 (27.6)	1,051 (23.9)	<0.001

* Mann–Whitney U test.

a This “Other” category was provided for those who may identify outside of the binary gender options.

### Dietary and lifestyle habits associated with tGERS

In multivariate analysis, the factors associated with tGERS included eating beyond fullness (odds ratio [OR] 1.383, confidence interval 95% [CI95%] 1.127–1.698), tight clothing (OR 1.627, CI95% 1.256–2.107), stress (OR 1.566, CI95% 1.363–1.800), and insomnia (OR 1.321, CI95% 1.129–1.546) (Table 2).

**Table 2:** Factors associated with troublesome gastroesophageal reflux symptoms in univariate multivariate analysis.

Predictors	Univariable			Multivariable		
	OR	95% CI	*p*	OR	95% CI	*p*
Female	1.135	0.989–1.304	0.072	1.104	0.951–1.281	0.192
Age > 30	1.307	1.150–1.484	<0.001	1.376	1.200–1.578	<0.001
Body mass index	1.037	1.018–1.057	<0.001	1.027	1.006–1.048	0.012
Post-graduate	1.249	1.077–1.448	0.003	1.108	0.949–1.295	0.195
Office work	1.209	1.064–1.373	0.003	1.156	1.011–1.320	0.033
Staying up late after midnight	1.101	0.962–1.259	0.162	1.019	0.880–1.180	0.802
Eating within 2 h before bed	1.122	0.991–1.271	0.069	1.032	0.907–1.175	0.631
Eating beyond fullness	1.565	1.288–1.901	<0.001	1.383	1.127–1.698	0.002
Tight clothing	1.792	1.395–2.302	<0.001	1.627	1.256–2.107	<0.001
Stress	1.703	1.500–1.934	<0.001	1.566	1.363–1.800	<0.001
Insomnia	1.564	1.358–1.802	<0.001	1.321	1.129–1.546	<0.001

### Characteristics of tGERS and management patterns

The characteristics of tGERS are presented in Table 3. Regurgitation was more prevalent than heartburn (49.3 and 20.3%, respectively). A total of 30.5% (716/2350) of participants experienced both symptoms, and 62.3% (1,465/2350) of them experienced tGERS for more than 1 year.

**Table 3:** Clinical characteristics of patients with troublesome gastroesophageal reflux symptoms (tGERS) (*N* = 2,350).

Clinical characteristics of troublesome gastroesophageal reflux symptoms (tGERS)	*N* (%)
**tGERS**	
Regurgitation	1,158 (49.2)
Heartburn	476 (20.3)
Regurgitation and heartburn	716 (30.5)
**Frequency of tGERS in the last 7 days (days)**	
0	354 (15.1)
1	675 (28.7)
2–3	735 (31.3)
4 or more	586 (24.9)
**The time tGERS happened**	
During the day	1,089 (46.3)
At night	462 (19.7)
Both during the day and at night	799 (34.0)
**First time experience with tGERS**	
< 1 month	244 (10.4)
1 to 6 months	321 (13.7)
6 to 12 months	320 (13.6)
2 years to 5 years	800 (34.0)
More than 5 years	665 (28.3)

**Figure 2:**
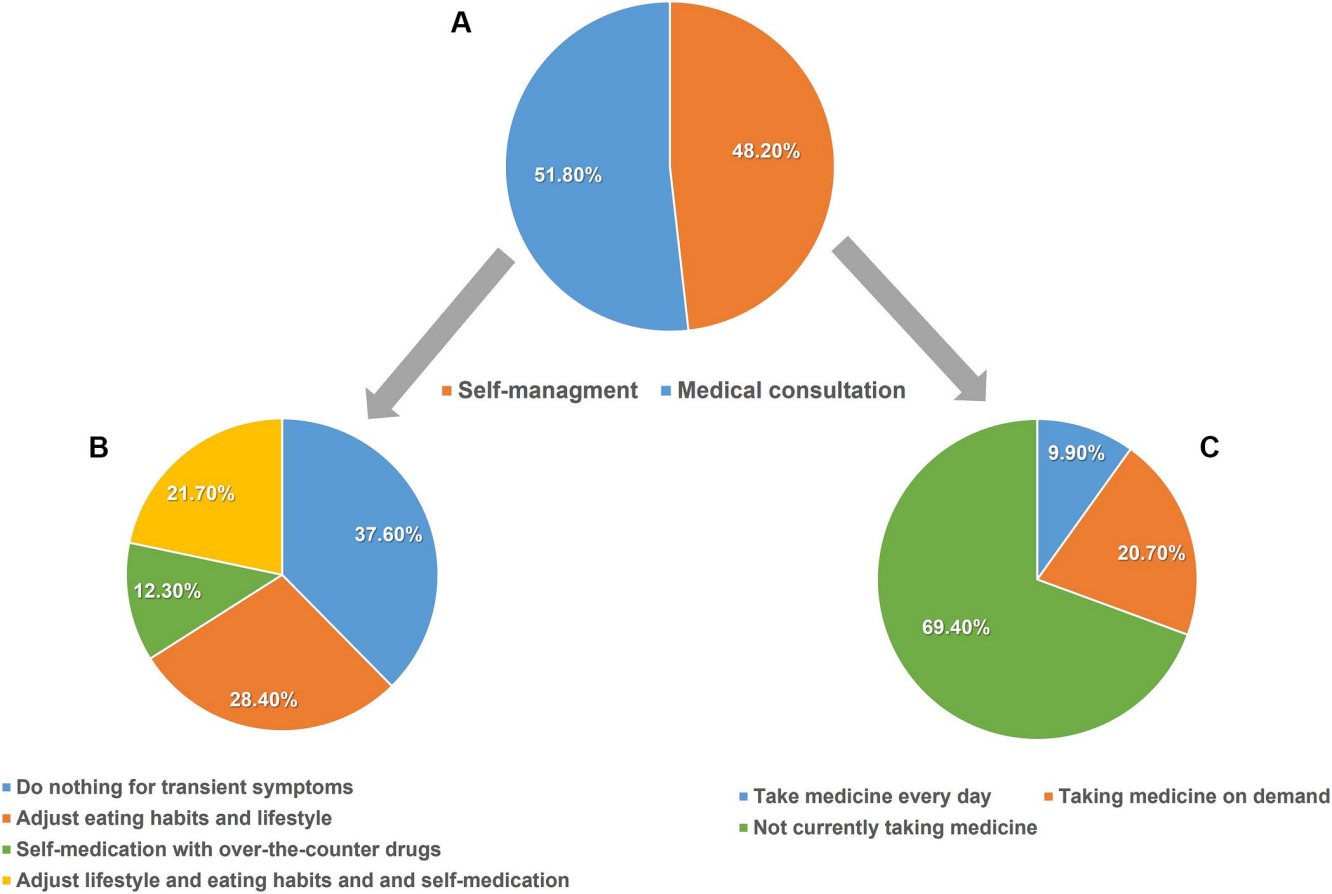
Participants’ management methods for tGERS. **(A)** Proportions of self-management and medical consultation of participants presenting with tGERS (*N* = 2,350). **(B)** Self-management methods in participants with self-management of tGERS (*N* = 1,133). **(C)** Current medical treatment in participants seeking medical consultation (*N* = 1,217).

**Figure 3:**
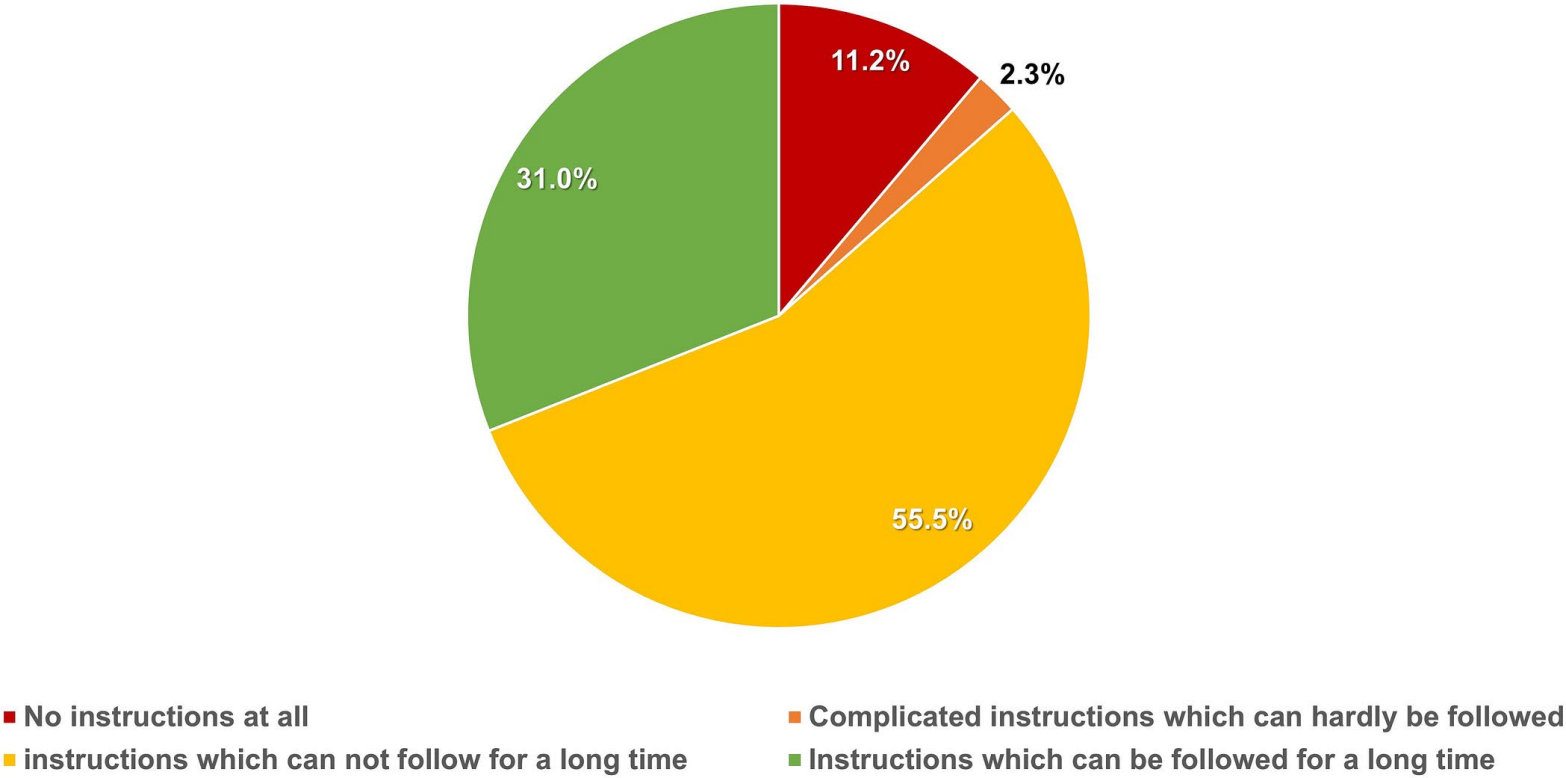
Physicians’ instructions regarding lifestyle and diet modifications in participants seeking medical consultation (*N* = 1,217).

The participants’ management methods for tGERS are presented in Figure 2. Only 1,217 (51.8%) sought medical consultation, while 1,133 (48.2%) chose self-management methods, including dietary and lifestyle modifications, self-medication, or applying both methods (28.4, 12.3, and 21.7%, respectively). A total of 11.3% (137/1217) of participants who sought medical consultation reported not receiving any advice about dietary and lifestyle modification from their physicians. For those who sought medical consultation and received advice on dietary and lifestyle modification, 89.0% (961/1080) reported that tGERS was reduced to a satisfactory level. However, 55.5% (675/1217) reported that they could not follow the advice for the long term (Figure 3).

### Dietary and lifestyle habits reported as triggers in subjects with tGERS

**Figure 4:**
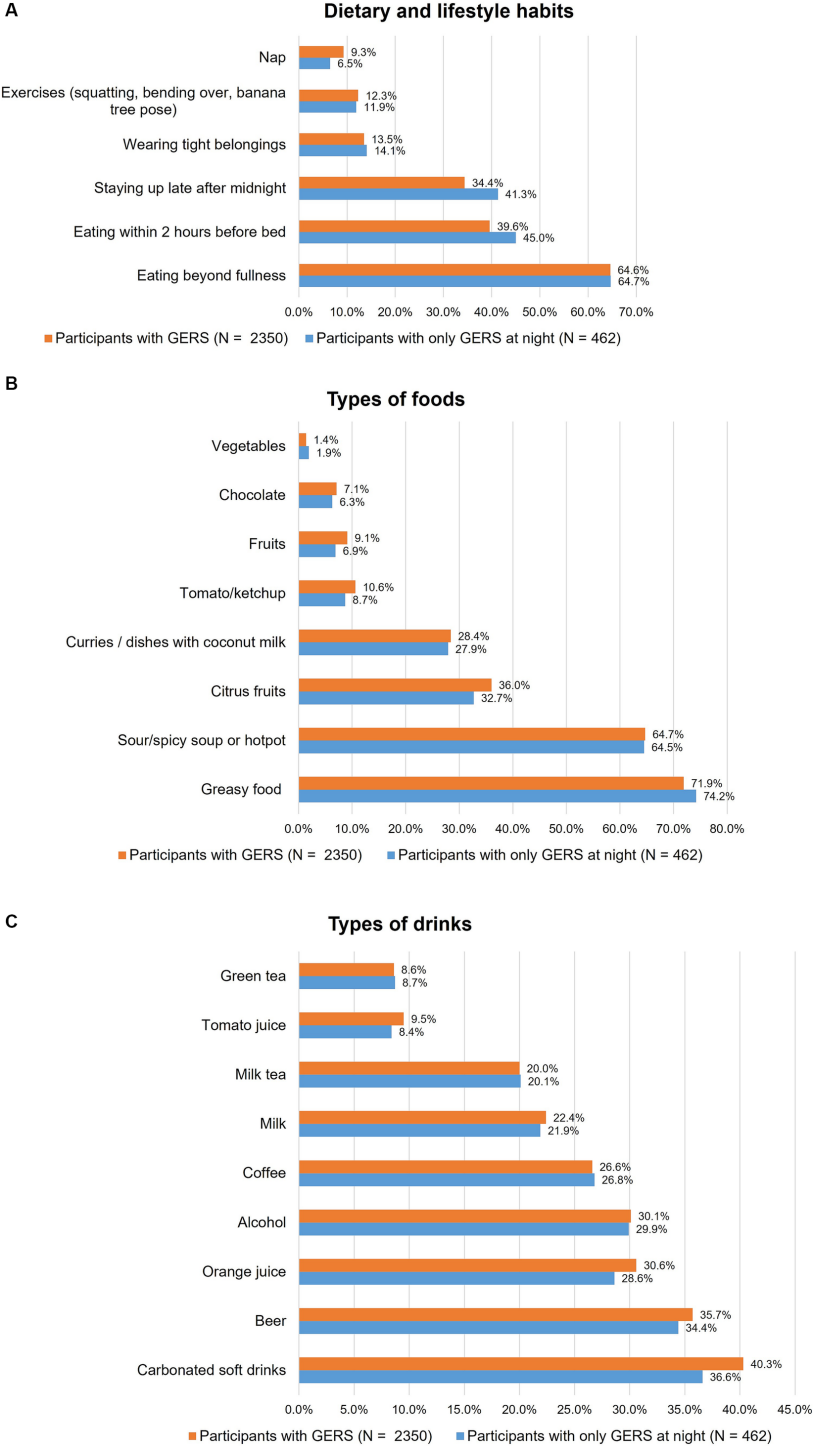
Factors that associated with troublesome gastroesophageal reflux symptoms (tGERS) and nocturnal tGERS: **(A)** Dietary and lifestyle habits, **(B)** Food types, and **(C)** Drink types.

The dietary and lifestyle habits that associated with tGERS are shown in Figure 4. Eating beyond fullness was the most frequently reported habit that triggered tGERS (64.6%). Interestingly, although a short meal-to-bed time and staying up late after midnight were not risk factors for tGERS, they were two common factors that triggered tGERS in symptomatic participants, particularly those with nocturnal reflux symptoms. Regarding foods that triggered tGERS, the top three most common foods were greasy foods (71.9%), sour/spicy soups (64.7%), and citrus fruits (36.0%). In terms of beverages, carbonated soft drinks were at the top tGERS trigger (40.3%), followed by beer (35.7%) and orange juice (30.6%).

## Discussion

This is the first study investigating the dietary and lifestyle habits associated with tGERS among the Vietnamese population. A recent systematic review showed that the risk factors for tGERS varied greatly across populations (9). Our study showed that eating beyond fullness, wearing tight clothing, stress, and insomnia were significantly associated with tGERS. This is consistent with prior studies in Asia. Meanwhile, a few other factors, which were found to be related to tGERS in previous studies, such as smoking, skipping breakfast, eating within 2 h before going to bed, and staying up late, were not associated with tGERS in our study (14–16). The differences among studies can be due to a variety of factors. First, it may stem from the diversity of diets and lifestyles in differing ethnicities as well as countries worldwide. Second, the definitions and measurements of dietary habits vary widely across studies. In addition, the dietary and lifestyle habits in each individual may change over time. Therefore, it is challenging to assess these factors accurately. Third, recall bias is an additional challenge that makes it difficult to obtain precise measurements.

When considering only the group of participants suffering from tGERS, we found that nearly half of them did not seek medical consultations but opted for various self-management approaches for their symptoms. This finding highlights the need to provide appropriate public health education on the self-management of tGERS. As relapse is the nature of GERD, it requires a long-term management plan in which dietary and lifestyle modifications are the mainstay of treatment (4, 7, 8). Our study showed that more than half of the participants with tGERS could not follow their physicians’ advice on dietary and lifestyle modifications. Therefore, identifying exactly the habits that associated with tGERS in a particular population is crucial. This helps avoid unnecessary modifications that may reduce the patient’s quality of life and lead to difficulty in their long-term adherence, hence symptom recurrence.

This study found that eating beyond fullness is the most common trigger of tGERS. Interestingly, although the habits of lying down within 2 h after meals and staying up late were not risk factors for tGERS when compared with asymptomatic participants, they were the two most common triggers for the onset of tGERS, especially at night, in symptomatic participants (Figure 4A). In a previous study conducted in Vietnam among pregnant women, we found that a short meal-to-bed time was the most important risk factor for tGERS (17). This is probably explained by a progressive decrease in the pressure of the lower esophageal sphincter and a decrease in gastric motility due to hormonal alterations in pregnancy (18). In another study, we found that subjects with abnormal gastroesophageal flap valves had significantly higher gastroesophageal reflux disease questionnaire (GERDQ) scores than those with normal flap valves (19). Therefore, the difference in findings in our current study may be partly elucidated by the variations in the functional or anatomic antireflux barrier across our participants. With the results of this study, we think that while short meal-to-bed time should be avoided in individuals with tGERS, such advice is probably unnecessary for primary prevention of tGERS in the general population.

Our study showed that foods and drinks that trigger tGERS in Vietnamese individuals are diverse. The most common foods were fatty or fried food, spicy soups, citrus fruits and curries, and dishes with coconut milk. For beverages, carbonated soft drinks were the top, followed by beer, alcohol, coffee, and orange juice. This result is similar to previous studies in other populations, but some differences exist. First, previous Asian studies did not find carbonated soft drinks to be associated with tGERS; in our study, this was the most common trigger drink (9). Meanwhile, these beverages were reported to trigger tGERS in many Western populations (9, 20). Second, there are differences in the associations between milk and coffee and tGERS in Asian countries. A study in India showed that occasional milk intake was a risk factor for GERD (21). A recent Taiwanese study reported that coffee added to milk or sugar was not associated with tGERS (22). Both of these studies measured the intake of these beverages in subjects with or without tGERS but were not based on subjective reporting by subjects with tGERS. Although these drinks were not risk factors for tGERS in our study, 26.6% of the participants who had experienced tGERS reported that coffee could trigger tGERS, and the figure for milk was 22.4%. Coffee had little to no effect on the lower esophageal sphincter pressure, but it might have direct irritant effects that trigger tGERS (23, 24). It has also been reported that high-fat milk and dairy products could worsen heartburn (25). The composition and volume of the drinks as well as the timing of drinking may contribute to the difference across studies.

Our study has some limitations. First, the questionnaire used in this study has not been well validated. Second, the assessment of some variables in the study was subjective depending on the patient’s perception, such as tight clothing and eating beyond fullness. In addition, some other factors, such as lack of physical exercise and alcohol consumption, have not been addressed. Third, when analyzing the foods or drinks that could worsen tGERS, the amount ingested, which was certainly related to the symptoms, has not been established. As there are a variety of foods and drinks, it is difficult to measure all in one study due to the overwhelming workload for participants. However, we think that the results of this study will be a premise for more in-depth studies on some of the factors associated with tGERS with more accurate and thorough measurements. Fourth, as the participants were mainly from urban areas, the findings of this study may not represent the entire Vietnamese population.

In conclusion, we reported the dietary and lifestyle habits associated with tGERS in Vietnamese for the first time. The findings of this study will serve as a basis for further studies on the primary prevention and nondrug management of tGERS in Vietnam.

## Data availability statement

The raw data supporting the conclusions of this article will be made available by the authors, without undue reservation.

## Ethics statement

The studies involving humans were approved by Ethics Committee in Biomedical Research of Nhan Dan Gia Dinh Hospital, Ho Chi Minh City, Viet Nam (number 011/NDGD-HDDD, signed on March 22, 2023). The studies were conducted in accordance with the local legislation and institutional requirements. The participants provided their written informed consent to participate in this study.

## Author contributions

DQ: Conceptualization, Data curation, Formal analysis, Funding acquisition, Investigation, Methodology, Project administration, Resources, Supervision, Validation, Visualization, Writing – original draft, Writing – review & editing. ML: Data curation, Formal analysis, Investigation, Methodology, Software, Validation, Writing – review & editing. PN: Data curation, Investigation, Writing – review & editing. UV: Investigation, Methodology, Writing – review & editing. CV: Investigation, Writing – review & editing.
