# Supplementary material for: Dietary and lifestyle factors associated with troublesome gastroesophageal reflux symptoms in Vietnamese adults
Source: Front Nutr. 2023 Nov 8;10:1280511. doi: 10.3389/fnut.2023.1280511 (PMC10663327; doi:10.3389/fnut.2023.1280511)
Supplement: Supplementary file 1 [file Data_Sheet_1.PDF]

SURVEY ON DIETARY AND LIFESTYLE FACTORS ASSOCIATED WITH TROUBLESOME GASTROESOPHAGEAL REFLUX SYMPTOMS AMONG VIETNAMESE ADULTS

Information Sheet and Informed Consent

Dear Sir/Madam,

Gastroesophageal reflux disease is a common health problem in Vietnam. This condition is closely related to dietary and lifestyle habits. This has been proven through many studies worldwide. Vietnamese people have different dietary and lifestyle habits compared to other populations. However, there are very few surveys on the impact of these habits on gastroesophageal reflux symptoms among Vietnamese adults. This survey is conducted to identify the dietary and lifestyle factors associated with these symptoms among Vietnamese adults, thereby contributing to community health improvement.

The survey will take approximately 5 - 10 minutes. We hope you can spend some of your time to participate and complete this survey. Your participation is entirely voluntary. All information will remain confidential. The results will be shared via popular domestic media outlets.

Thank you for your participation.

If you have any questions about this study, please contact the research team at: gerdinvietnam@gmail.com

On behalf of the research team:

Duc Trong Quach, MD, PhD (University of Medicine and Pharmacy at Ho Chi Minh City)

Cong Hong-Minh Vo, MD, PhD (Nhan Dan Gia Dinh Hospital)

Uyen Pham-Phuong Vo, MD, MSc (University of Medicine and Pharmacy at Ho Chi Minh City)

Mai Ngoc Luu, MD, MSc (University of Medicine and Pharmacy at Ho Chi Minh City)

Phong Van Nguyen, MD, MSc (Nhan Dan Gia Dinh Hospital)

By completing and submitting this survey, you consent to participate.

Do you agree to participate in the survey?

- ☐ Yes
- ☐ No *(If the participant chooses this, the survey ends)*

I. Demographic information

1. Your age:
    - ☐ Under 18 years old *(If the participant chooses this, the survey ends)*
    - ☐ 18 to 30 years old
    - ☐ 31 to 40 years old
    - ☐ 41 to 50 years old
    - ☐ 51 to 60 years old
    - ☐ Over 60 years old
2. Gender:
    - ☐ Male
    - ☐ Female
    - ☐ Other
3. Height (cm): …
4. Current weight (kg): ….
5. Highest educational level:
    - ☐ Primary
    - ☐ Secondary
    - ☐ High school
    - ☐ University/ college
    - ☐ Post-graduate
6. Domicile:
    - ☐ Urban
    - ☐ Rural
7. Current career:
    - ☐ Office work
    - ☐ Freelance trading/ business
    - ☐ Transportation - delivery (frequently moving on roads)
    - ☐ Housewife

- ☐ Agriculture/manual labor
- ☐ Student
- ☐ Retirement
- ☐ Other, specify …

II. Dietary and lifestyle habits

	Never	Rarely	Occasionally	Fairly Often	Very Often
1. How often do you stay up late after midnight?					
2. How often do you skip meals?					
3. How often do you eat (including snacks, fruits, or drink alcohol) **<u>within 2 hours before bed (nap or nighttime)?</u>**					
4. How often do you eat beyond fullness (continue eating even when feeling full)?					
5. How often do you wear tight clothing?					
6. How often do you smoke?					
7. How often do you feel stressed?					
8. How often do you suffer from insomnia?					

III. Reflux symptoms

1. **<u>In the past month</u>**, have you experienced any of the following discomforts?
    - ☐ A burning sensation in the middle chest, right behind the sternum (also called **heartburn**)
    - ☐ Regurgitation of sour fluid or food from the stomach to the throat/mouth (also called **regurgitation**)
    - ☐ Both of the above symptoms (**heartburn and regurgitation**)
    - ☐ NONE of the two symptoms *(If the participant chooses this option, the survey ends*)
2. How many days **in the past 7 days** have you had these troublesome reflux symptoms (heartburn, regurgitation)?
    - ☐ 0 *(If the participant chooses this option, the survey ends)*
    - ☐ 1 day

- 2 – 3 days
- 4 days or more

3. When do these troublesome reflux symptoms (heartburn and/or acid regurgitation) usually occur in the day?
    - During the day
    - At night
    - Both day and night
4. When was the first time you first experienced these troublesome reflux symptoms?
    - Recently, within the past month
    - 1 – 6 months ago
    - 6 months – 1 year ago
    - 1 – 5 years ago
    - Over 5 years
5. How do you usually manage these troublesome reflux symptoms?
    - Adjusting diet and lifestyle habits.
    - Self-medication with over-the-counter drugs.
    - Adjusting diet and lifestyle habits and self-medication.
    - Do nothing as the symptoms are transient and bearable.
6. Have you ever sought for medical consultation because of troublesome reflux symptoms?
    - Yes
    - No *(if this option is selected, proceed to question 9)*
7. If you've seen a doctor due to troublesome reflux symptoms, were you given instructions on lifestyle and diet modifications? How well did you follow it?
    - I wasn't given any instructions *(if this option is selected, proceed to question 9).*
    - The instructions were complicated, so they could hardly be followed.
    - I was given instructions and I could follow them for a long time.
    - I was given instructions but I couldn't follow them for a long time.
8. Did your reflux symptoms improve when you followed the doctor's dietary and lifestyle instructions?

- ☐ Yes
- ☐ No

9. Are you currently taking medication for reflux?
   - ☐ Yes, daily
   - ☐ Yes, but not regularly
   - ☐ No
10. Which of the following lifestyles triggers your troublesome reflux symptoms? (select all that apply)
    - ☐ Napping
    - ☐ Staying up late after midnight
    - ☐ Eating beyond fullness
    - ☐ Eating within 2 hours before bed
    - ☐ Specific exercises causing reflux (squatting, bending over, banana tree pose)
    - ☐ Wearing tight belongings
    - ☐ Other, specify: …
11. Which of the following foods triggers your troublesome reflux symptoms? (select all that apply)
    - ☐ Curries, dishes with coconut milk
    - ☐ Sour/spicy soup or hotpot
    - ☐ Greasy food
    - ☐ Fruits
    - ☐ Citrus fruits
    - ☐ Vegetables
    - ☐ Tomato/ketchup
    - ☐ Chocolate
    - ☐ Other (please specify): …
12. Which of the following beverages triggers you reflux symptoms? (select all that apply)
    - ☐ Orange juice

- ☐ Tomato juice
- ☐ Carrot juice
- ☐ Coconut water
- ☐ Milk
- ☐ Alcohol
- ☐ Beer
- ☐ Green tea
- ☐ Coffee
- ☐ Milk tea
- ☐ Carbonated soft drinks
- ☐ Other (please specify): …

THANK YOU FOR TAKING THIS SURVEY
